# A comparative study examining laparoscopic and open inguinal hernia repair in children: a retrospective study from a single center in China

**DOI:** 10.1186/s12893-020-00912-7

**Published:** 2020-10-19

**Authors:** Jie Liu, XiongWei Wu, WenLi Xiu, XiWei Hao, Jing Zhao, Bin Wei, Qian Dong

**Affiliations:** 1 Department of Pediatric Surgery, Affiliated Hospital of Qingdao University, Qingdao University, No. 16 Jiangsu Road, Shinan District, Qingdao, 266000 Shandong Province China; 2 Department of Pediatric Surgery, Yijishan Hospital of Wannan Medical College, Wannan Medical College, Wuhu, 246400 Anhui Province China; 3 Shandong Key Laboratory of Digital Medicine and Computer Assisted Surgery, Affiliated Hospital of Qingdao University, Qingdao University, Qingdao, 266000 Shandong Province China

**Keywords:** Laparoscopy, Open surgery, Inguinal hernia, Child, Minimal invasive

## Abstract

**Background:**

Pediatric inguinal hernia (PIH) is a common disease in children. Laparoscopic hernia repair (LHR) has developed rapidly in recent years, but there are still different opinions compared with traditional open hernia repair (OHR). The purpose of this study was to compare the advantages and disadvantages of LHR and OHR in the treatment of pediatric inguinal hernia.

**Methods:**

We performed a retrospective review of all children (< 14 years) who underwent repair of inguinal hernia in the pediatric surgery center of the Affiliated Hospital of Qingdao University from January 2015 to December 2015. We collected the medical records of all the children and analyzed the clinical characteristics, operation-related information and follow-up.

**Results:**

In the OHR group, 202 cases underwent unilateral inguinal hernia repair, and 43 cases underwent bilateral inguinal hernia repair. In the LHR group, 168 cases underwent unilateral inguinal hernia repair, and 136 cases underwent bilateral inguinal hernia repair. There was a significant difference in the operation time between the two groups, but there were no significant differences in postoperative hospitalization time and incidence of ipsilateral recurrent hernia between the two groups. The incidence rates of metachronous contralateral hernia (MCH) and surgical site infection in LHR group were significantly lower than those in the OHR group.

**Conclusion:**

Our study shows that compared with OHR, LHR has the advantages of concealed incision, minimal invasiveness, reduced operation time, detection of contralateral patent processus vaginalis, and reduced incidence of MCH. In conclusion, LHR is safe and effective in the treatment of pediatric indirect inguinal hernia.

## Background

Pediatric inguinal hernia (PIH) is a common disease in children, with incidence rates ranging from 0.8 to 4.4% [1]. The main reason is congenital patent processus vaginalis (PPV). High ligation of the hernia sac can achieve satisfactory results [2]. Traditional open hernia repair (OHR) has been implemented in the clinic for many years, which has the characteristics of simple operation and strong popularity [3]. Some scholars believe that it is necessary to open the inguinal canal, which causes postoperative pain, and it is easy to damage spermatic vessels and the vas deferens [4]. However, laparoscopic hernia repair (LHR) has the advantages of having a concealed scar, being minimally invasive, and having the ability to detect the contralateral PPV. Many authors think that LHR may gradually replace OHR and become the main surgical method for PIH [5, 6]. At the same time, we also noticed that some scholars still have disputes about the operation time and the recurrence rate of hernia after LHR [1]. Therefore, we retrospectively analyzed the clinical and follow-up data of laparoscopic treatment of PIH and further compared the advantages and disadvantages of laparoscopic treatment of PIH to provide certain reference for clinical treatment.

## Methods

We performed a retrospective review of all children (< 14 years) who underwent repair of inguinal hernia in the pediatric surgery center of the Affiliated Hospital of Qingdao University from January 2015 to December 2015. All the information was approved by the ethics committee of the Affiliated Hospital of Qingdao University. In this study, the parents of all patients signed a preoperative informed consent form and agreed to participate in the accompanying scientific research. We did not disclose any personal information of the children when collecting the data. In this study, our senior pediatric hernia surgeons explained to the family members of the children the operation methods of the two techniques, the appearance of the incision after the operation and the possible complications of the operation, so that the parents of the children could choose the operation method they are willing to accept.

This study did not include cases of incarcerated hernia and other diseases requiring simultaneous surgical treatment. Since the PIH surgery is an elective operation, to avoid the influence of pneumoperitoneum on intraoperative anesthesia in young children, the operating age of children undergoing LHR in our center is generally controlled at more than 3 months.

## Clinical data

The characteristics of the child were achieved by consulting the medical records during hospitalization. Demographic data included sex and age at the time of surgery. Data on clinical features included the location of the hernia, the surgical method performed by the child, and the laterality of inguinal hernia diagnosed preoperatively and postoperatively.

### Surgical methods

**Fig. 1:**
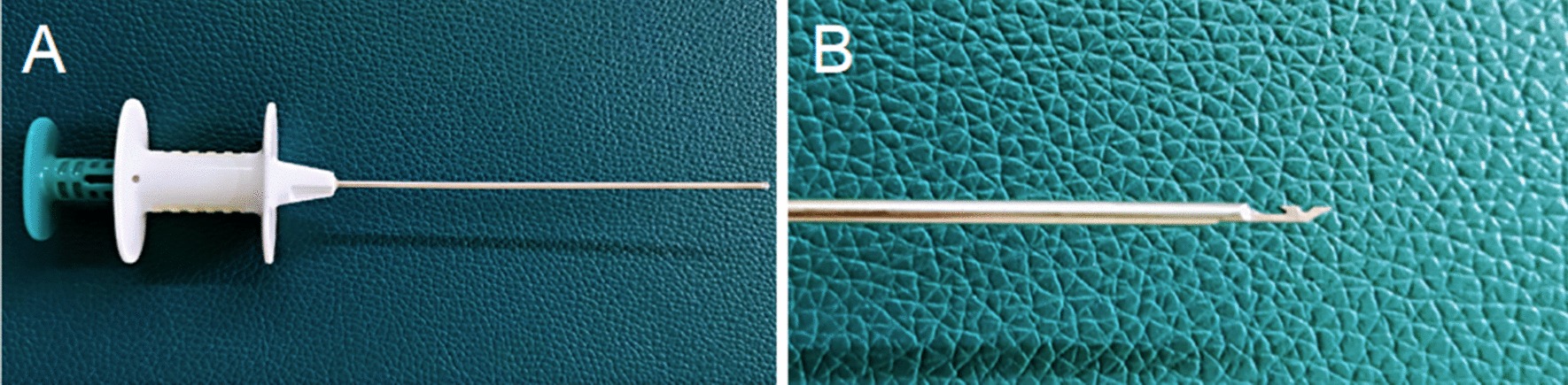
The two-hooked hernia needle apparatus. **a** The appearance of the apparatus. **b** The magnified image of the two slots in the core distal end

**Fig. 2:**
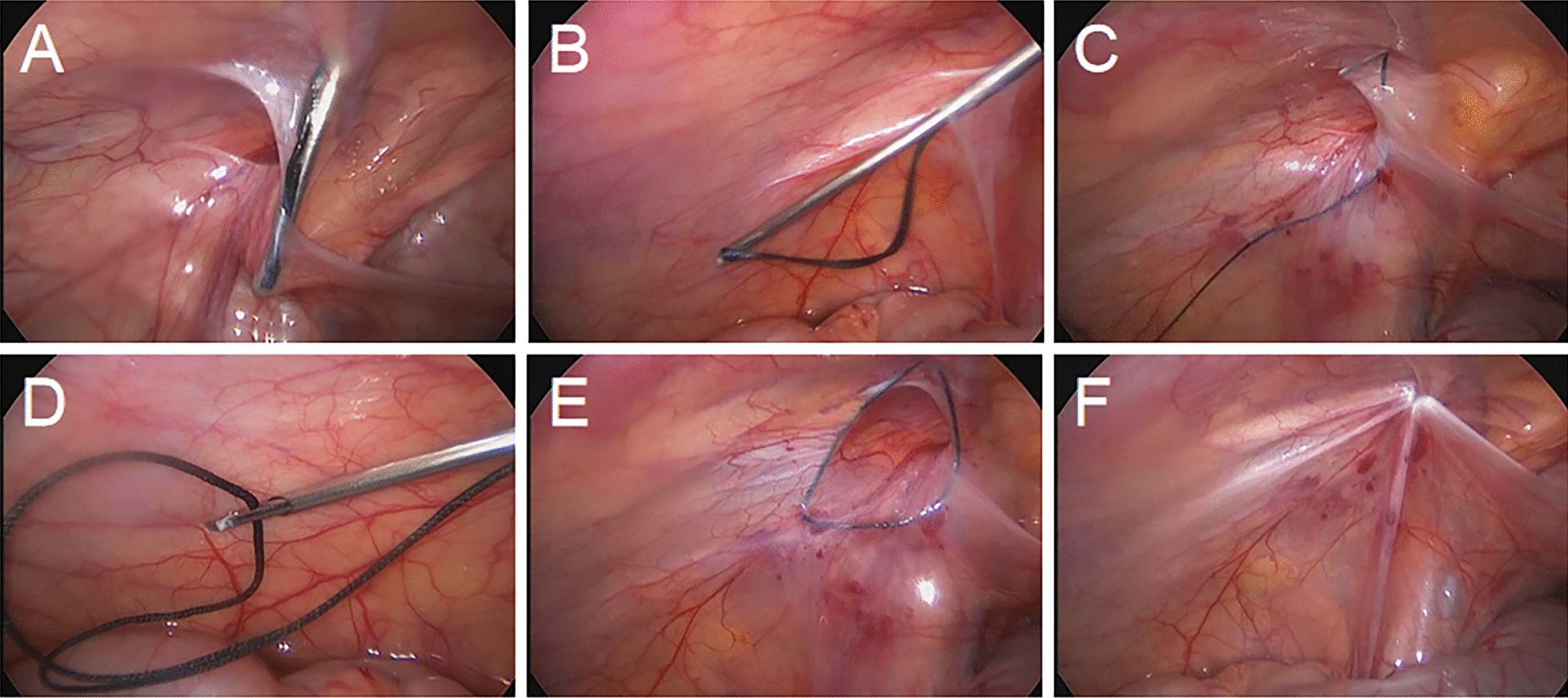
Images of single-port laparoscopic percutaneous extraperitoneal closure operation steps with the two-hooked hernia needle apparatus. **a** The needle enters the abdominal cavity around the inner side of the hernia ring and passes between the peritoneum and the vas deferens. **b** After the hernia needle passes through the spermatic vessels, the silk thread is retained in the abdominal cavity with the help of a lens. **c** The needle then goes around the outside of the ring and into the abdominal cavity. **d** The hernia needle hooks the preset silk thread and takes it out of the abdominal cavity. **e** The thread is tightened and tied under the skin. **f** The internal hernia ring is closed

**Fig. 3:**
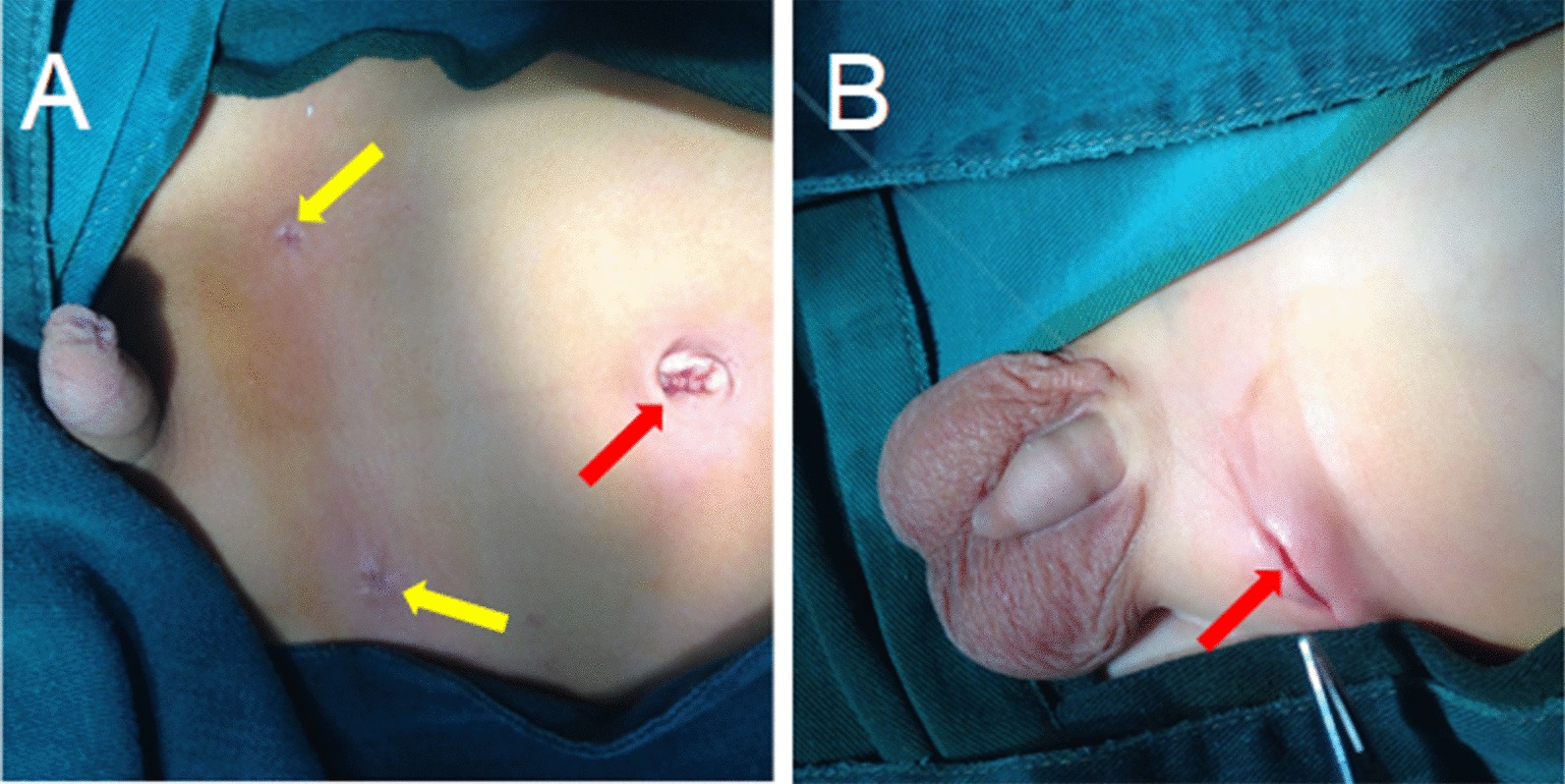
Comparison of incisions between laparoscopic hernia repair and open hernia repair. **a** The incision of laparoscopic inguinal hernia repair. The red arrow refers to the umbilical incision, and the yellow arrow refers to the needle hole left after bilateral inguinal hernia repair by the hernia needle puncture. **b** The open inguinal hernia repair incision. The red arrow points to the groin area approximately 1.5 cm along the abdominal transverse line incision

In the LHR group, laparoscopic percutaneous extraperitoneal closure was performed with a two-hooked hernia needle (Fig. 1), which was produced by Xiamen Surgaid Medical Equipment Co., Ltd. of China (Patent No. ZL 2013 20013865.2). The internal structure and use method of the hernia needle device were detailed in Li et al. [7]. All patients were given general anesthesia. The patients were asked to urinate and defecate before operation, and supine position was taken after anesthesia. A 5-mm diameter Trocar was placed through a curved incision on the lower edge of the umbilicus, and a 30° laparoscopic lens was placed. After entering the abdominal cavity, the organs in the abdominal cavity were routinely explored, the closure of bilateral internal rings was observed, and the intraoperative inguinal hernia was diagnosed again. Under close monitoring under laparoscopy, the skin was punctured with a knife tip or syringe needle at the lower abdominal transverse line corresponding to the inner ring of the affected side, and the 2-0 silk thread hook was hung in the shallow groove in front of the two-hooked hernia needle. The hernia needle was inserted into the abdominal cavity from the anterior abdominal wall of the body surface of the inner ring. When the hernia needle reaches the vas deferens and spermatic vessels, the surgeon can use a syringe connected to the tail to inject normal saline into the extraperitoneal space to separate the extraperitoneal space, to reduce the damage to the vas deferens and spermatic vessels. With the help of the laparoscopic lens, the silk thread is retained in the abdominal cavity. Then, the hernia needle slowly retracts into the extraperitoneal space along the original route, but it cannot retreat to the muscular layer and cannot come out from the puncture point. After that, it goes under the peritoneum on the outside of the inner ring and enters the abdominal cavity again through the puncture gap of the peritoneum. The head end of the silk thread reserved in the abdominal cavity is drawn out and taken out of the abdominal cavity. Finally, the silk thread is ligated to the hernia sac and tied in vitro, and the knot is located under the skin (Figs. 2, 3a).

In the OHR group, an approximate 1.5-cm transverse incision along the dermatoglyph was taken in the inguinal area of the affected side, and the subcutaneous tissue was incised layer by layer. The hernia sac was separated and found and then ligated with silk thread at the position of the inner ring. Attention should be paid to protecting the vas deferens from damage when cutting the hernia sac (Fig. 3b).

### Follow-up schedule

In this study, regular outpatient or inpatient follow-up and telephone follow-up were utilized to accomplish the follow-up, which included ipsilateral recurrent hernia, contralateral metachronous hernia, surgical site infection and other surgical complications. The patients were followed up until September 2019.

### Statistical Analysis

All statistical analyses in this study were carried out using the R programming language, version 3.6.0 (R Foundation), in which a P-value < 0.05 was considered statistically significant.

## Results

From January 2015 to December 2015, all procedures were successfully performed for 245 patients who underwent OHR and for 304 patients who underwent LHR in our center. The demographic and clinical characteristics of the two groups are shown in Table 1. There was no significant difference in baseline characteristics between the two groups before operation.

**Table 1:** Demographic data of the OHR and LHR groups before operation

Characteristics	OHR	LHR	t (χ^2^) value	P value
Sex (No.)			χ^2^ = 0.045	0.832
Male	217	271		
Female	28	33		
Median age (Months)	15(3 ~ 154)	15(3 ~ 304)	t = 0.057	0.954
Laterality (Preoperative, No.)			χ^2^ = 0.027	0.869
Unilateral	202	249		
Bilateral	43	55		

*OHR* open surgery inguinal hernia repair; *LHR* laparoscopic inguinal hernia repair; *No.* number

**Fig. 4:**
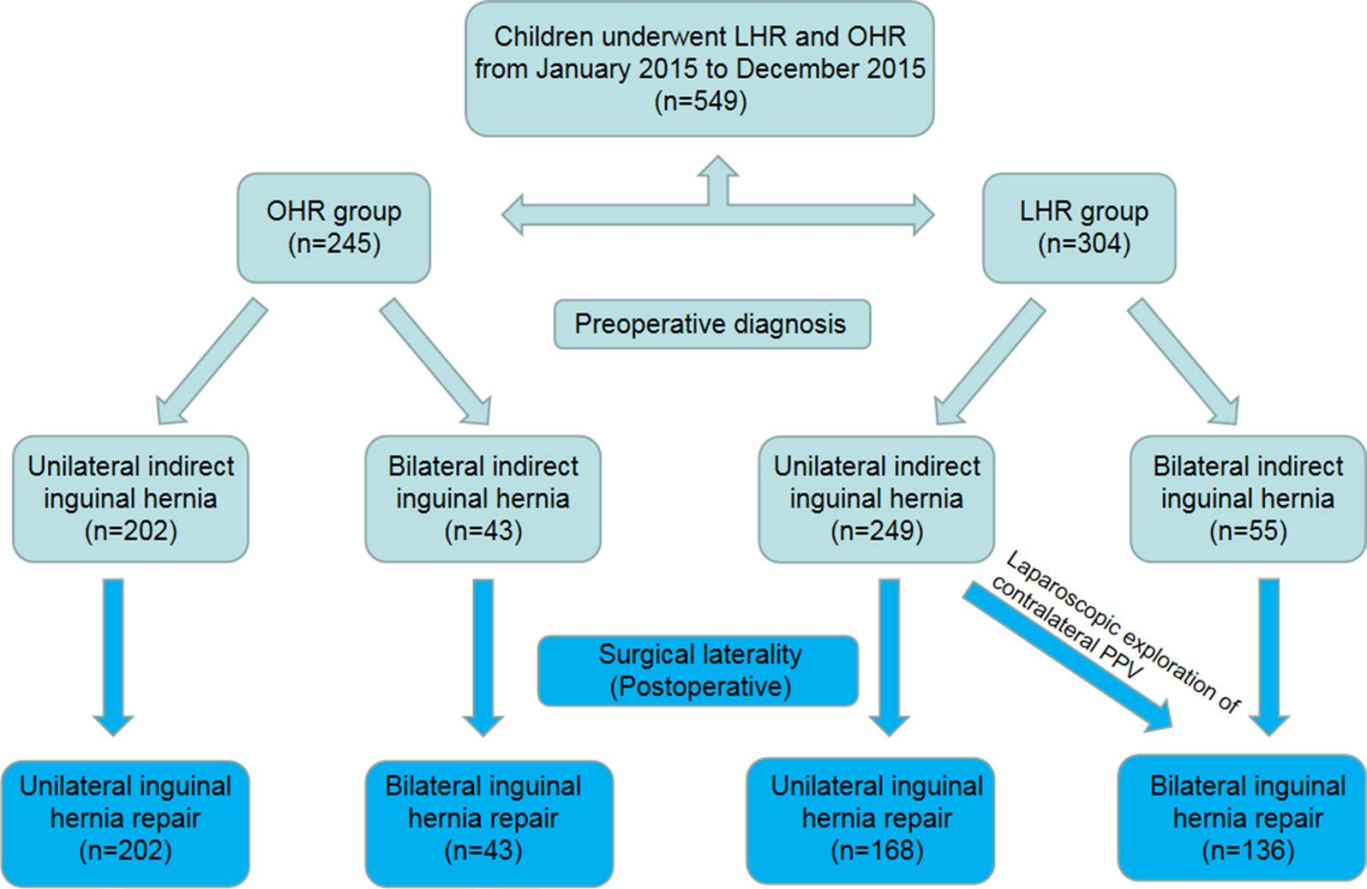
Identification of patients who underwent open surgery inguinal hernia repair and laparoscopic inguinal hernia repair

The postoperative diagnosis, operation time and postoperative hospitalization time of the two groups are shown in Table 2. In the OHR group, 202 cases underwent unilateral inguinal hernia repair, and 43 cases underwent bilateral inguinal hernia repair. In the LHR group, 168 cases underwent unilateral inguinal hernia repair, and 136 cases underwent bilateral inguinal hernia repair. There was a significant difference in surgical laterality between the two groups (P < 0.001). In the LHR group, 81 of 249 children with unilateral inguinal indirect hernia diagnosed preoperatively were found to have contralateral PPV, with a rate of 32.5%. All of them were ligated simultaneously (Fig. 4).

**Table 2:** Comparisons of operation site, operation time and postoperative hospitalization time between the two groups

Characteristics	OHR	LHR	t (χ^2^) value	P value
Operation time (min)				
Unilateral	37.52 ± 5.13	16.17 ± 4.24	t = 43.792	< 0.001
Bilateral	61.60 ± 8.50	19.92 ± 4.55	t = 30.797	< 0.001
Laterality (Postoperative, No.)			χ^2^ = 45.629	< 0.001
Unilateral	202	168		
Bilateral	43	136		
Postoperative hospitalization time (days)				
Unilateral	1.49 ± 0.44	1.46 ± 0.43	t = 0.810	0.418
Bilateral	1.49 ± 0.43	1.42 ± 0.43	t = 0.925	0.356

*OHR* open surgery inguinal hernia repair; *LHR* laparoscopic inguinal hernia repair; *No.* number

**Fig. 5:**
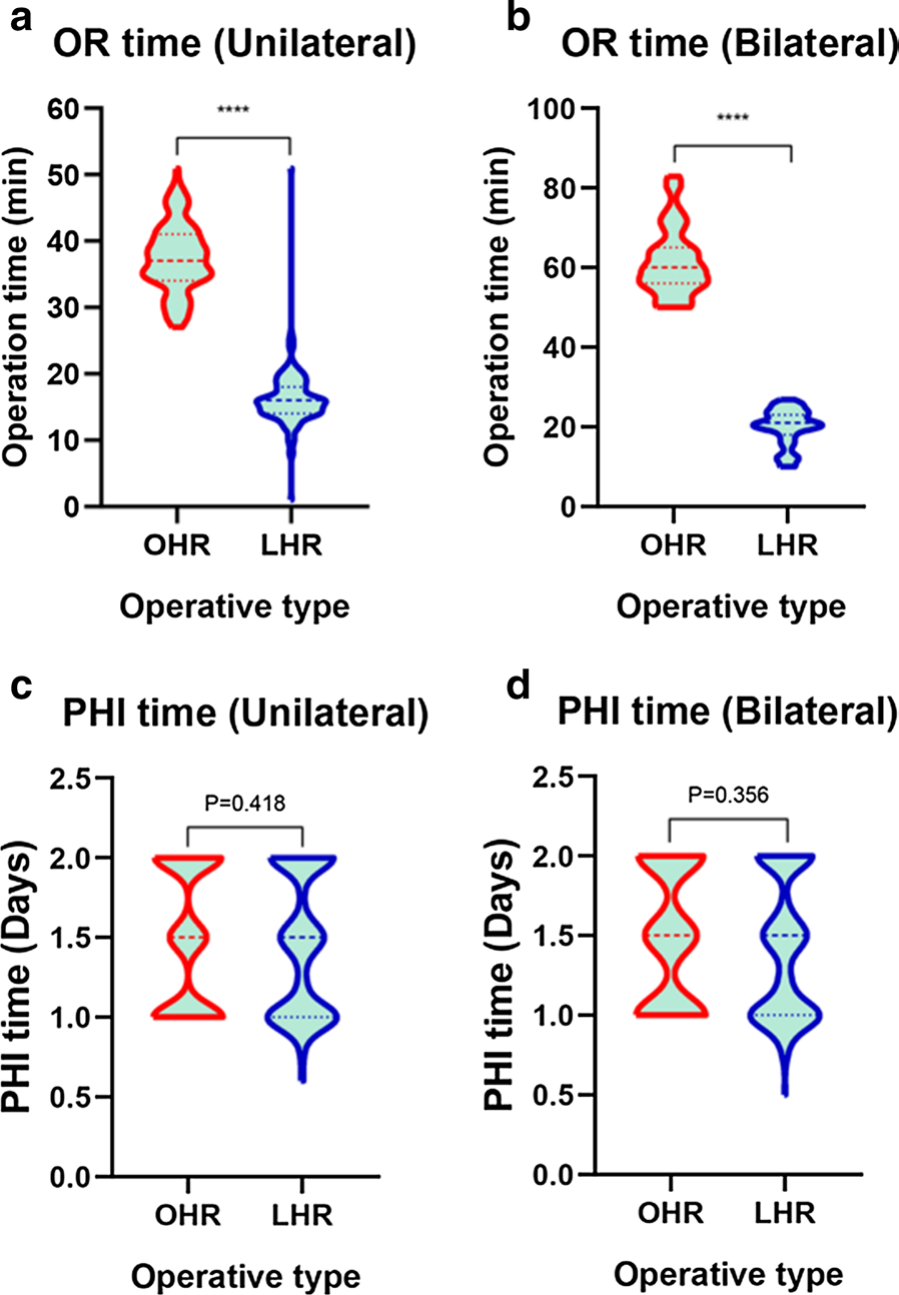
Violin plots of the two groups of children were produced by t-test of operation time and postoperative hospitalization according to the actual operation laterality (unilateral or bilateral). **a** There was a significant difference in unilateral operation time between the two groups. ****P < 0.001. **b** There was a significant difference in bilateral operation time between the two groups. ****P < 0.001. **c** There was no significant difference in postoperative hospitalization time between the two groups after unilateral inguinal hernia repair. P = 0.418. **d** There was no significant difference in postoperative hospitalization time between the two groups after bilateral inguinal hernia repair. P = 0.356

The operation times of unilateral and bilateral operations in the OHR group were significantly longer than those in the LHR group (P < 0.001) (P < 0.001) (Fig. 5), but there was no significant difference in postoperative hospital stay between the two groups (Table 2 and Fig. 5).

The incidence rates of hernia recurrence and postoperative complications in OHR group and LHR group are shown in Table 3. In this study, there was no significant difference in the incidence of ipsilateral hernia recurrence between the two groups (P = 0.610). The incidence of metachronous contralateral hernia (MCH) in the OHR group was significantly higher than that in the LHR group (P < 0.001). The surgical site infection rate in the OHR group was higher than that in the LHR group (P < 0.001).

**Table 3:** Postoperative complications in the OHR and LHR groups

Complications	OHR	LHR	χ^2^	P value
Ipsilateral recurrent hernia	14/245 (5.7%)	15/304 (4.9%)	0.259	0.610
Contralateral metachronous hernia	28/202 (13.9%)	1/249 (0.4%)	33.406	< 0.001
Surgical site infection	2/245 (0.8%)	1/304 (0.3%)	33.581	< 0.001
Hydrocele (male)	6/217 (2.8%)	1/271 (0.4%)	4.893	0.027
Scrotal swelling (male)	17/217 (7.8%)	1/271 (0.4%)	21.382	< 0.001
Iatrogenic ascent of the testis (male)	1/217 (0.5%)	0/271	1.251	0.263
Testicular atrophy (male)	0/217	0/271	–	–

*OHR* open surgery inguinal hernia repair; *LHR* laparoscopic inguinal hernia repair

Among male children, the incidence of scrotal swelling in the OHR group (7.8%) was significantly higher than that in the LHR group (0.4%). The difference was statistically significant (P < 0.001). The incidence of postoperative hydrocele in the OHR group was higher than that in the LHR group (P = 0.027). Postoperative iatrogenic ascent of the testis occurred in only 1 case in the OHR group and in 0 cases in the LHR group, and there was no difference between the two groups (P = 0.263). In this study, there were no cases of testicular atrophy.

## Discussion

With the rapid development of endoscopic technology, laparoscopic surgery is widely accepted by pediatric surgeons because it can reduce the obvious skin incision of traditional surgery and improve the aesthetic effect of surgery [8]. In this study, there were almost no surgical scars in the LHR group because the umbilicus was a hidden natural scar, but there were still 1.5-cm obvious surgical scars in the inguinal area in the OHR group. However, whether LHR can be better than OHR in every aspect, such as operation and postoperative complications, and can provide more benefits to children is still controversial [9, 10].

Some studies have reported that LHR increases the operation time compared with OHR [9], while a prospective study of 64 children by Igwe et al. [10] reported that there was no difference between laparoscopic operation time and open operation time. Our results showed that the operation time of the LHR group was significantly lower than that of the OHR group, whether unilateral or bilateral. We believe that laparoscopic technology does not need to dissect the inguinal canal. The hernia needle can directly enter the abdominal cavity under the lens of the monitor to complete the hernia repair, thus greatly saving operation time.

LHR can detect MCH and contralateral PPV simultaneously, which is a significant advantage of laparoscopic surgery over open surgery [11]. However, it is still controversial whether the contralateral PPV should be ligated at the same time [4]. Some scholars believe that only 10% of the contralateral PPV patients will further develop into MCH, and there is no need for treatment [12]. However, more scholars believe that the contralateral PPV should be ligated and closed at the same time to prevent the MCH that may occur at any time in the later stage [7]. Because simultaneous ligation will neither increase the risk nor cause an additional injury to the patient, it cannot be ignored in the operation. In this study, we ligated and closed the MCH and contralateral PPV in the LHR group. All patients had no other complications, and the incidence of MCH was significantly reduced compared with the OHR group.

In general, the recurrence rate of PIH after surgery is very low [8]. A meta-analysis by Alzahem suggested that the recurrence rate of hernia after LHR was higher than that of traditional OHR [13]. Interestingly, Yang et al. [14] carried out the same meta-analysis. In his paper, he found that the results of two studies in the included literature were similar to those of Alzahem [13, 14]. However, three studies have shown that laparoscopic techniques reduce the recurrence rate after surgery, and two other studies have shown no difference between the two. In a prospective study conducted by Abd-Alrazek et al. [15], it was concluded that no hernia sac resection during laparoscopic surgery did not increase the recurrence rate of hernia. In this study, to prevent the recurrence of the huge hernia with the diameter of the inner ring greater than 1.5 cm, it is necessary to re-ligate and cover the medial umbilical fold on the same side as the inner ring peritoneum [7]. In the actual operation, we realize that when the coverage of the medial umbilical fold is strengthened, the peritoneum in most cases has been swollen; thus, special attention should be paid to avoid vas deferens ligation, and normal saline can be injected if necessary. For older children with giant hernia, their self-control ability is poor, and they may have too much activity after getting up; thus, time of physical activity after operation should be appropriately delayed. As of the time of publication, there was no recurrence after the treatment of giant hernia by this method. We found that if indirect inguinal hernia with hydrocele occurs in children, after high ligation of the hernia sac, we can puncture and drain the hydrocele with a syringe needle under the light of the laparoscopic lens. In our study, there was no difference in the recurrence rate of prime sites between the two groups, but it could reduce the incidence of postoperative hydrocele in male children. Therefore, we believe that the decrease of the incidence of postoperative hydrocele may be related to more complete and firm ligation under laparoscopy, and the determinants of postoperative hernia recurrence may be multifaceted.

Some scholars have carried out a meta-analysis and think that the incidence of incision infection after laparoscopic surgery is increased [16]. However, in our study, 1 case of incision infection in the laparoscopic group was umbilical incision infection, while 2 cases in the open group were infected with inguinal incision. All cases were cured after dressing change and oral antibiotic treatment. There were no cases of needle sinus granuloma in the two groups until the publication date, and the incidence of postoperative infection in the LHR group was lower than that in the OHR group. Our experience is that no matter what kind of operation method is adopted, aseptic operation technology must be strictly carried out, the incision tissue of umbilical or inguinal region should be protected as far as possible, and repeated puncture caused by trocar slipping of the umbilical region should be avoided. In the LHR group, the hernia needle we used was a two-hooked needle. After the silk thread is passed through the vas deferens and spermatic vessels and is put into the abdominal cavity, the hernia needle must not withdraw to the muscle layer under the abdominal wall or outside the incision; it should only exit to the extraperitoneal space. This can not only ensure that the silk thread will not tie other tissues in the ligation, but will also greatly reduce or avoid the possibility of a ligation line. We believe that this may reduce the incidence of granuloma.

Our study also has a few limitations. For example, the cases from a single center may have a minor impact on our analysis results. The reason for the slightly higher recurrence rate in this study may be related to the fact that the cases included in the study come from a single center. There may be some differences between the statistical results and the multi-center study. We will continue to collect follow-up data in the later study to further analyze the possible causes of recurrence. In addition, the follow-up time was too short to compare the effects of two surgical methods on children's reproductive behavior in adulthood.

## Conclusion

Our study shows that compared with OHR, LHR has the advantages of concealed incision, minimal invasiveness, reduced operation time, detection of contralateral patent processus vaginalis, and reduced incidence of MCH. In conclusion, LHR is safe and effective in the treatment of pediatric indirect inguinal hernia.

## Data Availability

All data is contained within the manuscript. The datasets used and analysed during the current study available from the corresponding author on reasonable request.

## Abbreviations

PIH: Pediatric inguinal hernia
PPV: Patent processus vaginalis
OHR: Open hernia repair
LHR: Laparoscopic hernia repair
MCH: Metachronous contralateral hernia
